# Reply to Lee and Elliott: Changes of bonding upon crystallization in phase change materials

**DOI:** 10.1073/pnas.2405294121

**Published:** 2024-04-29

**Authors:** Jean-Yves Raty, Christophe Bichara, Carl-Friedrich Schön, Carlo Gatti, Matthias Wuttig

**Affiliations:** ^a^Condensed Matter Simulation, Université de Liège, Sart-Tilman B4000, Belgium; ^b^Aix-Marseille University, CNRS, UMR7325, Marseille 13288, France; ^c^Institute of Physics 1A, Rheinisch-Westfälische Technische Hochschule Aachen, Aachen 52074, Germany; ^d^Consiglio Nazionale delle Richerche, Istituto di Scienze e Tecnologie Chimiche “Giulio Natta”, Milano 20133, Italy; ^e^Istituto Lombardo Accademia di Scienze e Lettere, Milano 20121, Italy; ^f^Peter-Grünberg-Institute (PGI 10), Forschungszentrum Jülich, Jülich 52428, Germany

**Fig. 1.**
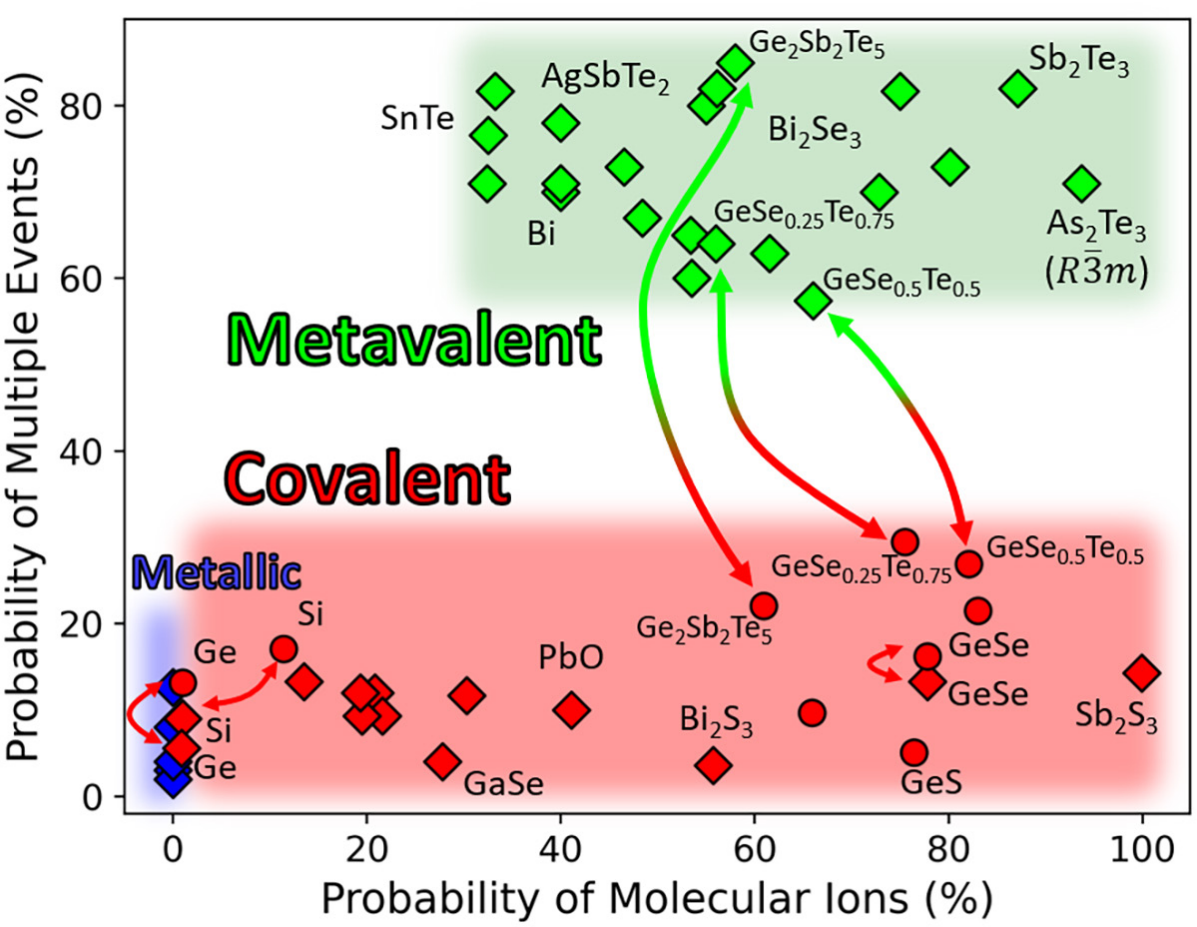
Changes of bonding upon crystallization (arrows). Crystals are characterized by diamonds, glasses by circles. Bonding is characterized by two parameters (PMI and PME), which quantify the bond rupture in atom probe tomography (4, 5).

In their letter, Lee and Elliott question the existence of a distinct class of glass-forming materials (1) which are at variance with Zachariasen's conjecture, i.e., form non-Zachariasen Glasses (NZGs) (2). Zachariasen postulated in 1932 that oxide glasses such as SiO_2_ have the same short-range order as the corresponding crystal. Zachariasen suspected that this similarity would be the consequence of a similarity in chemical bonding (2). We have confirmed this claim for SiO_2_ and chalcogenides like GeSe and GeSe_2_ (3). However, there are other chalcogenides like GeTe and related compounds, which do not obey his conjecture. This has been demonstrated for both properties and quantum chemical bonding descriptors, which change significantly for these NZGs upon crystallization (3). Further support is depicted in Fig. 1, showing data for the bond rupture obtained by atom probe tomography (4, 5). The two crucial parameters, defined in (5), hardly change upon crystallization for GeSe, Si, and Ge, as expected for Zachariasen glasses (short red arrows), while for the three NZGs (Ge_2_Sb_2_Te_5_, GeSe_0.25_Te_0.75_, and GeSe_0.5_Te_0.5_) pronounced changes in bonding are found (long green—red arrows).

Lee and Elliott assert that the atomic arrangement is very similar in the crystalline and glassy state (1). However, at variance with their claims, avoiding the use of cut-off distances, figure 2 in ref. 3 evaluates differences between glass and crystal in PCMs. This confirms that these differences are much more pronounced in NZGs than in ZGs, in line with other studies (6–8). The close interrelation between differences in atomic arrangement and differences in bonding is mandatory to explain pronounced differences in, e.g., optical properties, a hallmark of phase change materials (6).

Finally, in ref. 1, it is argued that bonding in c-PCMs is hypervalent (vs covalent in the glass). This seems to create a contradiction. If the bonding in the two phases is identical (as stated in ref. 1), how can it be differentiated into two classes (hypervalent vs. covalent)? Hypervalent bonding characterizes a scenario where four electrons hold together three atoms. If hypervalent bonding would prevail in crystalline GeTe, where the same bonding motif exists in three almost orthogonal directions, 12 electrons would be required to form these bonds (9). However, GeTe does not have 12 valence electrons, rendering hyperbonding (3c–4e bonding) impossible. Instead, bonding in these chalcogenides is primarily governed by 6 p-electrons (6). For an atomic arrangement with six nearest neighbors, this immediately explains why the bonding is apparently electron-deficient (2c–1e, or 3c–2e) bonding, as supported by detailed quantum chemical calculations (8, 10, 11).

The concerns expressed in ref. 1 appear to be based on a conceptual ambivalence, where changes of bonding and atomic arrangement are reported but considered irrelevant. Instead, our paper (3) and this comment demonstrate that most phase change materials indeed are at variance with Zachariasen’s conjecture, i.e., they significantly alter their atomic arrangement, bonding, and properties upon crystallization.
